# The β-catenin/CBP-antagonist ICG-001 inhibits pediatric glioma tumorigenicity in a Wnt-independent manner

**DOI:** 10.18632/oncotarget.15934

**Published:** 2017-03-06

**Authors:** Maria Wiese, Neele Walther, Christopher Diederichs, Fabian Schill, Sebastian Monecke, Gabriela Salinas, Dominik Sturm, Stefan M. Pfister, Ralf Dressel, Steven A. Johnsen, Christof M. Kramm

**Affiliations:** ^1^ Division of Pediatric Hematology and Oncology, Department of Child and Adolescent Health, University Medical Center Goettingen, Goettingen, Germany; ^2^ Institute of Cellular and Molecular Immunology, University Medical Center Goettingen, Goettingen, Germany; ^3^ Transcriptome and Genome Analysis Laboratory (TAL), Department of Developmental Biochemistry, University Medical Center Goettingen, Goettingen, Germany; ^4^ Division of Pediatric Neurooncology, German Cancer Research Center (DKFZ), Heidelberg, Germany; ^5^ Department of Pediatric Hematology and Oncology, Heidelberg University Hospital, Heidelberg, Germany; ^6^ Department of General, Visceral, and Pediatric Surgery, University Medical Center Goettingen, Goettingen, Germany

**Keywords:** ICG-001, pediatric high-grade glioma (pedHGG), Wnt/β-catenin signaling, CREB binding protein (CBP), cell cycle

## Abstract

Pediatric high-grade gliomas (pedHGG) belong to the most aggressive cancers in children with a poor prognosis due to a lack of efficient therapeutic strategies. The β-catenin/Wnt-signaling pathway was shown to hold promising potential as a treatment target in adult high-grade gliomas by abrogating tumor cell invasion and the acquisition of stem cell-like characteristics. Since pedHGG differ from their adult counterparts in genetically and biologically we aimed to investigate the effects of β-catenin/Wnt-signaling pathway-inhibition by the β-catenin/CBP antagonist ICG-001 in pedHGG cell lines. In contrast to adult HGG, pedHGG cells displayed minimal detectable canonical Wnt-signaling activity. Nevertheless, low doses of ICG-001 inhibited cell migration/invasion, tumorsphere- and colony formation, proliferation *in vitro* as well as tumor growth *in vivo/ovo*, suggesting that ICG-001 affects pedHGG tumor cell characteristics independent of β-catenin/Wnt-signaling. RNA-sequencing analyses support a Wnt/β-catenin-independent effect of ICG-001 on target gene transcription, revealing strong effects on genes involved in cellular metabolic/biosynthetic processes and cell cycle progression. Among these, high mRNA expression of cell cycle regulator *JDP2* was found to confer a better prognosis for pedHGG patients. In conclusion, ICG-001 might offer an effective treatment option for pedHGG patients functioning to regulate cell phenotype and gene expression programs in absence of Wnt/β-catenin signaling-activity.

## INTRODUCTION

Tumors of the central nervous system (CNS) are among the most common pediatric malignancies, and pediatric high-grade gliomas (pedHGG) including anaplastic astrocytoma (AA), glioblastoma (GBM), and diffuse midline glioma account for approximately 15-20% of pediatric CNS tumors. Despite major improvements in pediatric oncology, the prognosis for pedHGG patients still remains very poor with overall-survival rates of less than 15% [1].

Approximately 30-50% of pedHGG are characterized by specific mutations in genes encoding variants of histone 3 (most commonly H3.3 encoded by the *H3F3A* gene, or H3.1 encoded by the *HIST1H3A/B/C* genes). H3 K27M mutations in midline gliomas result in global chromatin changes and are associated with an even worse clinical outcome compared to their wildtype counterparts. H3.3 G34R/V mutations are markedly less frequent and are preferentially found in older children, adolescents, and young adults with hemispheric HGG with no obvious association with survival [2–5].

Several studies in adult HGG, which mostly carry wildtype H3, suggested a potential impact of the canonical Wnt/β-catenin signaling pathway in the acquisition of an aggressive GBM phenotype [6–8]. Therefore, the Wnt/β-catenin signalling pathway may potentially be important for the phenotype of pedHGG and especially those carrying H3.3 wildtype gene or H3.3 G34R/V alleles.

Upon canonical Wnt stimulation, the central component, β-catenin, becomes stabilized and enters the nucleus where it serves to recruit the transcriptional co-factor cAMP response element-binding protein (CREB) binding protein (CBP) to complexes containing TCF/Lef transcription factors. The level of canonical Wnt-pathway activity and the combination of β-catenin-bound transcriptional co-factors define the activation of specific subsets of target genes, e.g. Axin-related protein (*AXIN2*), CD44 (Indian Blood Group) and Bone Morphogenetic Protein 4 (*BMP4*) [9–18]. Transcriptional regulation by CBP is crucial for the maintenance of stem cell potency [19, 20]. Accordingly, CBP/β-catenin interaction has also proven to not only be essential for self-renewal capacity, but also for resistance to chemotherapy [21] and for the inhibition of apoptosis [11] during tumorigenesis. Apart from its role in controlling transcription downstream of Wnt-signaling, CBP also regulates the expression of a broad range of other target genes [22] and possesses histone acetyltransferase (HAT) activity, which serves to open chromatin structure and recruit bromodomain-containing cofactors, thereby promoting gene expression [22–24].

The small molecule ICG-001 inhibits canonical Wnt-signaling activity by preventing interaction of β-catenin and CBP by binding the N-terminal amino acids 1-111 of CBP [11, 25]. Treatment with ICG-001 sensitizes adult glioma cells towards radiotherapy [26] and efficiently reduces tumor cell growth of different tumor entities, including pancreatic [27] and colon cancers [25]. Various clinical trials employ ICG-001 (or the clinical applicable equivalent drug PRI-724) for colon and pancreatic cancer or myeloid malignancies (NCT01606579; NCT01764477; NCT02413853).

To date, the relevance of CBP and the β-catenin/Wnt-signaling pathway in pedHGG cell models has not been explored. Therefore, this study aimed to investigate the efficacy of the small molecule inhibitor ICG-001 on pedHGG cell lines carrying wildtype H3 (UW479 and SF188) or an H3.3 G34V mutation (KNS42) to study H3 K27M-independent therapeutic effects of the canonical Wnt inhibitor ICG-001 on proliferation, migration, invasion, and stem cell-like properties.

## RESULTS

### ICG-001 inhibits glioma cell growth *in vitro*

**Figure 1:**
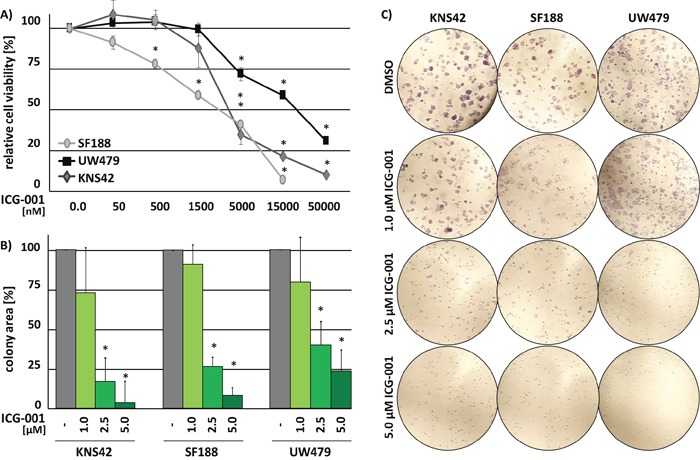
ICG-001 reduces pedHGG cell growth and clonogenicity in a dose-dependent manner **(A)** Cell viability of three pedHGG cell lines treated with increasing concentrations of ICG-001 for 72 h, as indicated (DMSO control = 100%; * p < 0.005). **(B)** Quantification and **(C)** brightfield images of colony formation assays after 8d of incubation at 20x magnifications. The relative colony area was determined in comparison to vehicle treated control cells (DMSO = 100%; * p < 0.05). Data are represented as mean of at least three biological replicates +/− SEM.

Treatment with ICG-001 reduced cell viability of pedHGG cell lines KNS42 (H3.3 G34V; derived from a pediatric GBM WHO grade IV), SF188 (H3.3 wildtype, derived from a pediatric GBM WHO grade IV) and UW479 (H3-wildtype, derived from an anaplastic astrocytoma WHO grade III) in a dose-dependent manner. KNS42 and SF188 cells were sensitive to lower concentrations of ICG-001 (KNS42 IC_50_ = 3 μM, SF188 IC_50_ = 2 μM) than UW479 cells (IC_50_ = 16 μM) (Figure 1A). ICG-001 treatment significantly inhibited the ability of all pedHGG cell lines to form colonies in a dose-dependent manner (Figure 1B and 1C). Colony formation was reduced to less than 10% in KNS42 and SF188 and 25% in UW479 cells. Thus, ICG-001 significantly inhibits growth of pedHGG cells.

### ICG-001 inhibits glioma self-renewal

**Figure 2:**
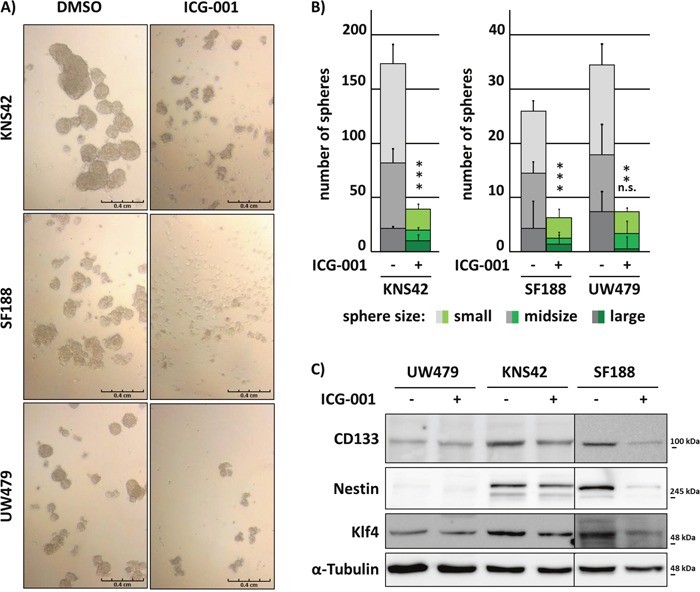
Glioma cell self-renewal is inhibited by ICG-001 ICG-001 inhibits KNS42, SF188 and UW479 cell-derived tumorsphere formation and reduces stem-like cancer cell associated marker protein expression in KNS42 and SF188 cells. **(A)** Brightfield images of tumorspheres and **(B)** quantification of sphere formation according to their size after 6 days of ICG-001 or vehicle control treatment, respectively. Large, midsize, and small spheres were defined as described in the “Material and Methods” section. **(C)** Western Blot analyses of whole cell lysates after 48 h incubation with ICG-001 or vehicle control, respectively. Membranes were incubated with antibodies detecting CD133, Nestin, Klf4 or α-Tubulin as loading control; (* p < 0.05; data are represented as mean +/− SEM).

Since the ability of invading tumor cells to self-renew is crucial for the formation of metastatic tumor foci within the infiltrated brain tissue, we determined the sphere-forming capacity of KNS24, SF188 and UW479 pedHGG cells after treatment with ICG-001. While SF188 and UW479 cells showed a lower sphere-forming capacity in comparison to KNS42 cells after 6 days, ICG-001 treatment significantly inhibited sphere formation in all cell lines. In addition, spheres formed by ICG-001-treated cells were significantly reduced in size (Figure 2A and 2B). To evaluate whether reduced sphere formation is accompanied by down-regulation of stem cell-associated markers indicating a specific effect by reduced stem cell-like ability, expression of glioma stem cell markers CD133, KLF4, and Nestin was assessed by Western Blot analysis. Consistent with decreased tumorsphere formation of UW479 compared to KNS42 and SF188 cells, basal protein expression levels of the investigated markers were lower in UW479 and not affected by ICG-001 treatment, while stem cell-associated marker protein expression was strongly reduced by ICG-001 in SF188 cells and slightly downregulated upon treatment in KNS42 cells (Figure 2C).

### ICG-001 suppresses motility of glioma cells

**Figure 3:**
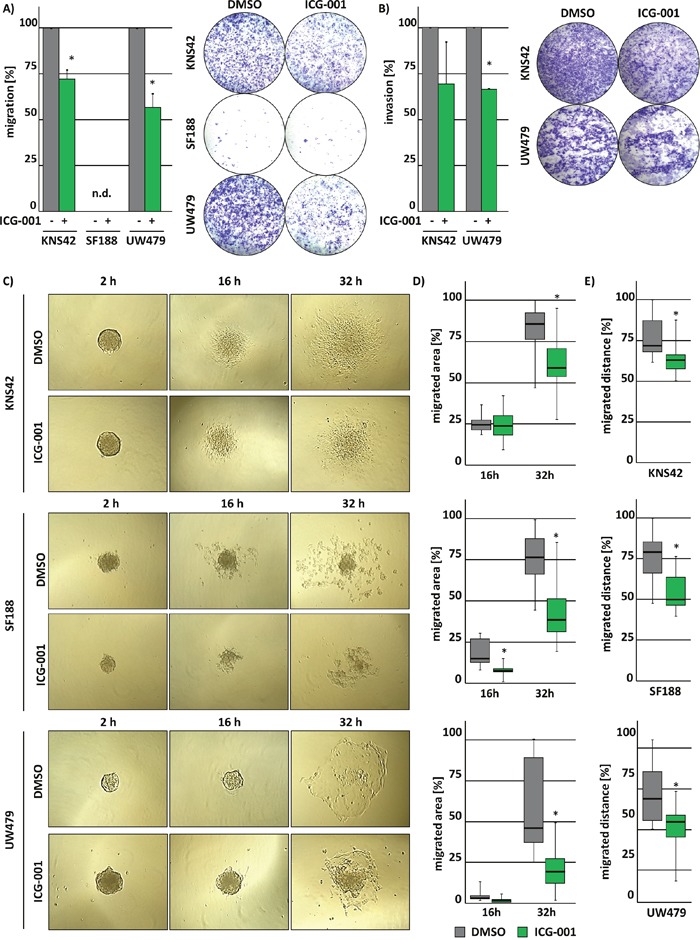
ICG-001 suppresses cell migration and invasion of glioma cells ICG-001 treatment reduces migration and invasion of pedHGG cells. Graphs show the quantification of **(A)** migrated and **(B)** invaded pedHGG cells after treatment with ICG-001. **(C-E)** Single tumorspheres derived from pedHGG cell lines in differentiation medium were monitored, and spheroid cell migration was assessed after 16 h and 32 h. **(C)** Representative brightfield microscopy images displaying migrating cells from single tumorspheres at different time points, as indicated. **(D)** Quantification of the migration area and **(E)** the migration distance; (* p < 0.05; data are represented as mean +/− SEM).

HGG belong to the most aggressive CNS tumors due to their ability to infiltrate into surrounding brain tissue. We therefore evaluated the effects of ICG-001 treatment on cell migration and invasion. Cell migration and invasion were significantly reduced after treatment with ICG-001 (Figure 3A and 3B). SF188 cells did not migrate as much as UW479 and KNS42 cells. Thus, invasion was not analyzed for SF188 cells after ICG-001 treatment.

In addition, investigating the migratory ability of KNS42-, SF188- and UW479-derived glioma stem-like cells from tumor spheres showed similar results, indicated by a significant reduction of the migration area (Figure 3C and 3D) and distance (Figure 3C and 3E). These observations demonstrate that ICG-001 inhibits the motility of pedHGG cells and their tumor sphere-derived equivalents.

### ICG-001 inhibits tumor growth *in vivo/ovo*

**Figure 4:**
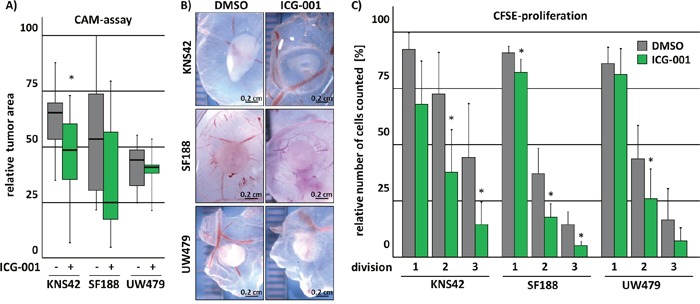
ICG-001 inhibits tumor growth *in vivo* ICG-001 treated pedHGG cells show diminished potential to form solid tumors on chorioallantoic membranes (CAM) *in vivo* and impaired proliferation as indicated by reduced cell-divisions *in vitro*. **(A-B)** PedHGG cell lines were pre-incubated with ICG-001 for 48 h; subsequently, equal cell numbers were placed on top of a CAM in an ECM-plaque and tumor growth was assessed by **(B)** brightfield images and **(A)** quantification of the grown tumor area. **(C)** Flow cytometric analysis of cell division by CFSE dilution reveals reduced proliferation following 24 h ICG-001 treatment of glioma cell lines that divided less efficiently (data are represented as mean of at least three biological replicates +/− SEM; * p < 0.05).

To evaluate if the *in vitro* effects of ICG-001 treatment on tumor growth of glioma cells can be recapitulated *in vivo*, we performed Chick Chorioallantoic Membrane (CAM) assays. To this end, pedHGG cells were incubated in a matrigel-plug with ICG-001 for 7 days on top of a CAM. After incubation, the area of the respective tumors was assessed (Figure 4A and 4B). Although only KNS42- but not UW479- and SF188-cell derived tumors displayed a significant decrease in tumor size, the tumor area deriving from pedHGG cells was overall reduced after exposure to ICG-001 in comparison to control cells (Figure 4A). While we observed invading tumor cells in the CAM independent of treatment conditions (data not shown), tumors derived from UW479 and KNS42 cell lines and subsequently treated with ICG-001 appeared to be more translucent in comparison to the densely packed vehicle treated tumors (Figure 4B). Treatment with ICG-001 led to impaired proliferation of all pedHGG cells as detected by marked reduction of cell divisions using CFSE-proliferation assays (Figure 4C), whereas no effect on Caspase-3-induced apoptosis or cell cycle arrest were observed (Supplementary Figure 1). In summary, KNS42, SF188 and UW479 cell-derived tumor growth is moderately inhibited *in ovo*, most likely through an inhibitory effect on cell proliferation.

### Wnt/β-catenin signaling is not essential for pedHGG proliferation

**Figure 5:**
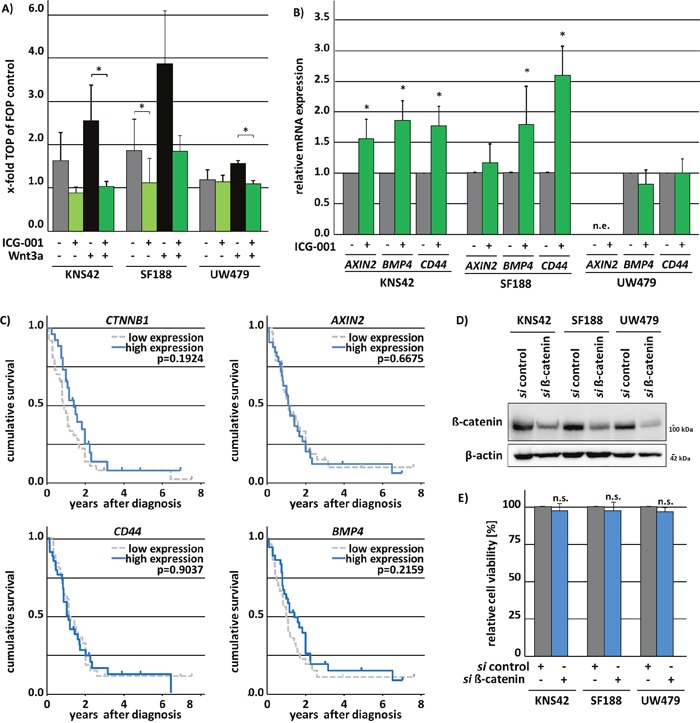
ICG-001 variably affects β-catenin/Wnt-signaling in pedHGG cell lines ICG-001 treatment inhibits Wnt3a-induced canonical Wnt-signaling in pedHGG cell lines and upregulates canonical Wnt-target gene expression although a downregulation would have been expected. **(A)** TOP/FOP Luciferase reporter gene assay of glioma cell lines after Wnt3a treatment to induce canonical Wnt-signaling and ICG-001 to inhibit β-catenin/CBP nuclear function. **(B)** qPCR-analysis of Wnt/β-catenin target genes *AXIN2, BMP4*, and *CD44* in KNS42, SF188 and UW479 pedHGG cell lines after 48h ICG-001 treatment. (data are represented as mean of at least three biological replicates +/− SEM; * p < 0.05; n.d., not detectable). **(C)** Kaplan-Meier survival analysis with the respective p-values of log rank analyses of 61 pediatric patients with GBM in dependence of gene expression levels (high/≥ median gene expression versus low/<median gene expression) indicate no impact of Δ-catenin (*CTNNB1*), *AXIN2, BMP4*, and *CD44* gene expression on pediatric patients survival with the following patient numbers per group: CTNNB1: low = 36, high = 25; CD44: low = 29, high = 32; AXIN2: low = 32, high = 29; BMP4: low = 29, high = 32. **(D)** Western Blots show the efficient knock down of Δ-catenin using whole cell lysates of pedHGG cells after siRNA mediated knock down of Δ-catenin. Membranes were incubated with antibodies detecting Δ-catenin or Δ-actin as loading control. **(E)** Cell viability of three pedHGG cell lines, treated with 50 pM *si* Δ-catenin for 72 h in comparison to control *si*RNA-treated cells, as indicated (*si* control = 1; n.s. not significant with p > 0.05; data are represented as mean of four independent, biological replicates +/− SEM).

To evaluate whether ICG-001 functions by inhibiting Wnt/β-catenin pathway activity in pedHGG cells, the TOP/FOP Luciferase-reporter system was used. Canonical Wnt-reporter gene activity was very low in KNS42 and SF188 cells (TOP/FOP ratio < 2) and undetectable in UW479 cells (TOP/FOP ratio = 1) (Figure 5A). To test whether canonical Wnt-signaling activity can be induced by simulation with Wnt and if this effect can be inhibited by ICG-001 treatment, pedHGG cells were simultaneously subjected to Wnt3a in combination with ICG-001 or vehicle co-treatment, respectively. Canonical TOP/FOP reporter-activity was induced in KNS42 and SF188 cells (TOP/FOP ratio < 2.5) and also elevated in UW479 (TOP/FOP ratio < 1.5) by Wnt3a. Simultaneous treatment with ICG-001 resulted in significantly lower reporter gene activity compared to vehicle control in pedHGG cell lines, confirming that the compound indeed inhibits β-catenin-mediated transcriptional activation (Figure 5A). In contrast, qPCR analysis of well-characterized β-catenin target genes *AXIN2, CD44*, and *BMP4* indicated no effect of ICG-001 on endogenous β-catenin target gene transcription in UW479, which lacked expression of *AXIN2* mRNA. In KNS42 and SF188 cells, expression of *AXIN2*, *CD44*, and *BMP4* was not inhibited, but rather significantly increased upon ICG-001 treatment (Figure 5B), suggesting a Wnt-independent effect of ICG-001 in pedHGG cells. Since previous studies demonstrated a correlation of canonical Wnt-signaling with the malignant phenotype of adult GBM, we examined a potential correlation between the expression of the β-catenin gene (*CTNNB1*) and its target genes *AXIN2*, *CD44* and *BMP4* with pedHGG patient survival data. Neither expression of *CTNNB1* itself nor of its target genes were correlated with clinical outcome (Figure 5C). To further investigate the emerging independent role of Δ-catenin in pediatric HGG cell lines, we assessed the cell viability of Δ-catenin deficient pedHGG cells after knockdown of Δ-catenin (Figure 5D). Confirming our previous results that indicated a redundant function of canonical Wnt signaling on pedHGG cells, reduced levels of Δ-catenin had no influence on cell viability (Figure 5E).

### ICG-001 regulates cell cycle-associated genes with putative clinical relevance

**Figure 6:**
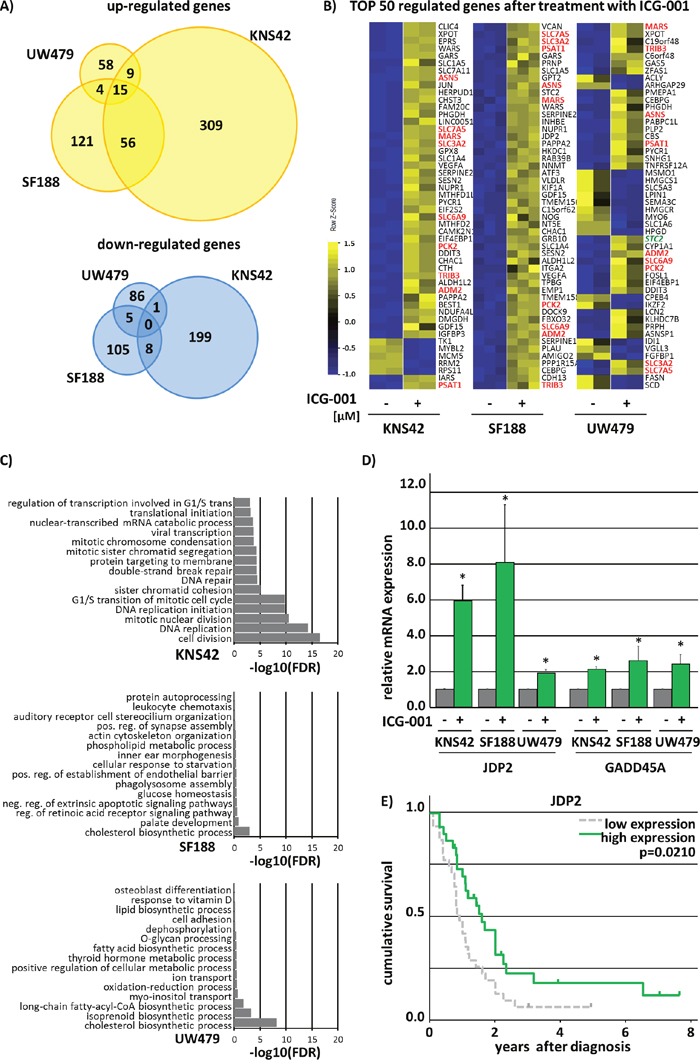
ICG-001 has differential effects on gene transcription without affecting the canonical β-catenin/Wnt-signaling pathway Analysis of significant (p < 0.05) 2-fold up- and down-regulated genes after 48h treatment with ICG-001 revealed no Wnt-phenotype in KNS42, SF188 and UW479 pedHGG cell lines. **(A)** Venn diagram analyses showing commonly and individually up- and downregulated genes by ICG-001. **(B)** Heat map illustrating the top 50 regulated genes, red marked genes are commonly changed in KNS42, SF188 and UW479 glioma cells. **(C)** GO-enrichment analyses depicting the fold discovery rate (FDR) of the top 20 enriched biological processes of all significantly 2-fold downregulated genes. **(D)** qPCR analyses proving the regulation of JDP2 and GADD25A by ICG-001 in three pedHGG cell lines, as indicated (DMSO control was set to 1; * p < 0.05; data are represented as mean of three independent, biological replicates +SEM). **(E)** ICG-001 regulates *JDP2* expression that is predictive for patients survival as shown by Kaplan-Meier survival analysis with the following patient numbers per group *JDP2*: low = 31, high = 30.

To investigate the molecular mechanisms underlying ICG-001 treatment in pedHGG we performed mRNA-sequencing (mRNA-Seq) after treating KNS42, SF188, and UW479 cells with ICG-001. A total of 593 transcripts showed significantly altered (2-fold change, p < 0.05) expression in KNS42 (385 up-regulated and 208 down-regulated), 314 transcripts in SF188 (196 up-regulated and 118 down-regulated) and 178 transcripts were significantly differentially expressed in UW479 (86 up-regulated and 92 down-regulated) (Figure 6A). The majority of the top 50 regulated genes in all cell lines concordantly showed increased expression upon inhibitor treatment (Figure 6B). Of note, treatment with ICG-001 induced significant up-regulation of the majority of various known β-catenin target genes in the three pedHGG cell lines (Supplementary Table 1).

To determine the potential biological effects of ICG-001 on pedHGG cells we performed gene ontology (GO) enrichment analyses using DAVID [28]. The top 15 GO-annotations of down-regulated genes by ICG-001 in UW479 and SF188 cells were associated with biosynthetic and metabolic processes with low false discovery rates (FDR -log10 < 9) (Figure 6C). Interestingly, GO-annotations for upregulated genes were also involved in biosynthetic and metabolic processes in UW479 and KNS42 line alone as well as when comparing all cell lines together (FDR -log10 < 6) (Supplementary Figure 2 data not shown). For all genes down-regulated after ICG-001 treatment in KNS42 cells GO annotations were related to cell cycle/cell division processes (FDR -log10 < 20) (Figure 6C and Supplementary Figure 2B and Supplementary Table 2).

Thus, we further analyzed ICG-001-induced expressional changes of cell cycle associated genes in more detail and found that ICG-001 strongly affects the expression of cell cycle regulators that play an essential role in the progression or the inhibition of all cell cycle phases, such as Cyclins (*CCN*) and Cyclin Dependent Kinases (*CDK*), suggesting dysregulation of all cell cycle phases upon ICG-001 treatment. While some of the down-regulated transcripts in KNS42 and SF188 cells were inversely regulated in response to ICG-001 in UW479 cells (Supplementary Figure 2A), commonly affected genes in both cell lines share a crucial role in cell cycle regulation: Cyclin-Dependent Kinase Inhibitor 1A (*CDKN1A*), Growth arrest and DNA-damage-inducible protein (*GADD45A*), and Jun Dimerization Protein 2 (*JDP2*) (Figure 6D and Supplementary Table 2). To evaluate whether ICG-001 regulated genes may have prognostic relevance, we performed Kaplan-Meier survival analyses in a cohort of pediatric HGG patients, revealing *JDP2* as predictor of survival in pedHGG patients. *JDP2* was strongly increased in all investigated pedHGG cell lines upon ICG-001 treatment (Figure 6D), and low *JDP2* expression was correlated with poor prognosis (Figure 6E). In summary, ICG-001 acts on glioma cells through Wnt/β-catenin-independent regulation of genes that are involved in cell cycle regulation and progression.

## DISCUSSION

The versatile role of β-catenin/Wnt signaling in controlling tumor cell properties such as self-renewal, motility, and radio- and chemo-resistance [29–31] has prompted the search for therapeutic strategies targeting this pathway. We observed inhibitory effects on pedHGG cell line proliferation, clonogenicity, self-renewal, migration, and invasion *in vitro* and tumor growth *in vivo* at lower concentrations of ICG-001 compared to previous studies in pancreatic cancer, multiple myeloma (MM), acute lymphoblastic leukemia, and colorectal cancer (CRC) [11, 27, 32, 33], suggesting a high sensitivity of pedHGG cells to ICG-001 treatment. In clinical trials, PRI-724, the clinically used equivalent to ICG-001, was applied in doses of up to 905 mg/m^2^/day (NCT01764477), corresponding to 55 μM ICG-001. Thus, the ICG-001 concentrations used in the present study *in vitro* may be well-achievable in patients. The comparably low doses of ICG-001 that resulted in pronounced effects on pedHHG cell behavior thus represent clinically relevant findings and further point to a specific effect of the compound rather than off-target effects.

The anti-tumor effects of ICG-001 in pedHGG cells presented here did not require canonical β-catenin/Wnt signaling pathway activation, although it cannot be completely excluded that ICG-001 treatment results in reduced β-catenin/CBP-dependent transcription in a small proportion of self-renewing pedHGG cells. However, besides this possible minimal β-catenin/Wnt-signaling activity we did not observe any effects on pediatric cell viability after knockdown of Δ-catenin confirming our finding of the Δ-catenin independency of pedHGG cells.

We also explored alternative mechanisms underlying the observed therapeutic efficacy of ICG-001 in pedHGG cells. Thus, we investigated the induction of caspase-3 dependent apoptosis by ICG-001, which had been previously reported in MM cells together with parallel upregulation of Noxa (*PMAIP1*) and other apoptosis-related genes. In the present study, *PMAIP1* was downregulated, and no effect on caspase-3 mediated apoptosis was observed. In addition, while we did observe proliferation defects following treatment, we did not reveal any significant effects on cell cycle phase distribution in pedHGG cells upon ICG-001 treatment. This is in contrast to previous reports on ICG-001-mediated induction of G1 cell-cycle arrest in other cancer cell lines [27, 32]. In pancreatic cancer cells, the ICG-001-mediated effect on cell cycle had been linked to genes particularly involved in G0/S1-phase transition: Cell Division Cycle25A (*CDC25A*), S-Phase Kinase-Associated Protein 2 (*SKP2*) and Cyclin dependent Kinase Inhibitor1A (*CDKN1A*) were all downregulated upon ICG-001 treatment; *CDKN1A* was identified as direct target of CBP [27]. ICG-001 treatment of SF188 resulted in less remarkable effects regarding the expression of cell-cycle-associated gene compared to KNS42 cells. While UW479 showed partially opposite effects with regards to the findings in pancreatic cancer cells, the effects on KNS42 and SF188 cells were in line with those.

In KNS42 cells, ICG-001 treatment led to downregulation of various genes which are involved in the regulation of the cell cycle, e.g. *CDK1/4, MCM2-7 and -10, as well as CCNE1, E2, A2, and B2*. Since this downregulation of cell cycle-associated genes did not result in a distinct cell cycle phase shift in pedHGG upon ICG-001 treatment suggesting a more global, phase-independent effect on cell cycle regulation. It therefore seems likely that ICG-001 induces transcriptional changes that inhibit the progression of different or even all cell cycle phases, leading to impaired proliferation and tumor growth *in vitro* and *in vivo*. In line with this hypothesis, two genes were strongly upregulated in all pedHGG cell lines that diversely control cell cycle progression at different checkpoints, namely *Jun Dimerization Protein 2* (*JDP2*) and *Cyclin-Dependent Kinase Inhibitor 1A* (*CDKN1A*, p21). In UW479 and KNS42 cells the tumor suppressor *Growth Arrest DNA Damage* (*GADD45A*) gene was up-regulated. In response to cellular stress, Gadd45 inhibits G1/S- and G2/M-Phase [34, 35] as well as cellular migration and invasion [36] reflecting our observations on proliferation, migration, and invasion of pedHGG cells upon ICG-001 treatment. Gadd45 interacts with several proteins, such as β-catenin, CDK1, and p21 (*CDKN1A*) to perform its versatile functions [37–41], whereas JDP2 acts directly at the DNA-level mostly resulting in transcriptional inhibition [42–44]. JDP2 overexpression is associated with an inhibition of epithelial-to-mesenchymal transition [45] which is associated with tumor cell migration and invasion in carcinomas and may also be relevant to glioma cell migration and invasion [46].

Furthermore, JDP2 also represses cell-cycle progression through inhibition of *CCNA2* expression [34]. Thus, it appears likely that Wnt-independent effects of ICG-001 may at least partially be mediated through upregulation of p21, Gadd45A, and JDP2, resulting in strong impairment of tumor-specific features in pedHGG cells. This assumption is corroborated by our observation that high *JDP2* mRNA expression is correlated with improved overall survival. Our results further imply interference of ICG-001 with amino acid metabolism, possibly leading to reduced tumor cell growth and proliferation.

Since secondary effects upon ICG-001 treatment cannot be fully excluded, further investigations will be needed to ascertain whether the identified genes are indeed direct targets of CBP-mediated transcriptional regulation. Furthermore, while ICG-001 prevents the interaction of β-catenin and CBP by binding within amino acids 1-110 of CBP [47], it likely also affects binding to other transcription factors and/or co-factors. Such β-catenin-independent mechanisms may be responsible for the observed ICG-001-induced effects on cellular proliferation, migration, and invasion. However, the mechanisms by which ICG-001 interferes with CBP function still remain to be fully elucidated and will be part of future investigations.

In summary, ICG-001 inhibits proliferation, clonogenicity, migration, and invasion in pedHGG cell lines, potentially via upregulation of *JDP2* and *GADD45A*. In addition, high *JDP2* expression is associated with a better prognosis in pediatric HGG patients. Thus, molecules such as ICG-001 and PRI-724, which directly target CBP, might represent a promising therapeutic option for the treatment of pedHGG.

## MATERIALS AND METHODS

### Cell culture and inhibitor treatment

The human pediatric glioblastoma cell line KNS42 was kindly provided by Pascal Johann (German Cancer Research Center (DKFZ) Heidelberg, Germany) and the pediatric anaplastic astrocytoma cell line UW479 and the pediatric glioblastoma cell line SF188 by Chris Jones (Institute of Cancer Research UK, London/Sutton, UK). Cells were cultured as described elsewhere [48]. Unless stated otherwise, adherent KNS42 and UW479 cells were treated with 2.5 μM, adherent SF188 cells were treated with 1 μM ICG-001 (Calbiochem, Darmstadt, Germany) dissolved in DMSO for 48h; the corresponding amount of DMSO (vehicle) was used as control.

### Cell viability assays

Cell viability was evaluated by 3-(4,5-dimethylthiazol-2-yl)-2,5-diphenyltetrazolium bromide (MTT)- assays. 1 × 10^5^ cells/ml were seeded in triplicates and adherent cells were treated with 10^0.7^ to 10^4.7^nM concentrations of ICG-001 and DMSO, as indicated, or a non-targeting pool or a pool of four different siRNAs targeting Δ-catenin (Dharmacon, Thermo Fisher scientific, further details below). After incubation for 72h, cells were incubated with 1 mg/ml MTT for 4h, lysed in DMSO-lysis buffer (33% DMSO, 5% formic acid, 62% isopropanol) and absorbance was measured at 550 nm. All experiments were performed with at least three independent biological cell passage replicates. P values < 0.05 were considered as significant and determined by student's t-tests.

### *si*RNA-mediated knockdown of Δ-catenin

For Western Blot analyses cells were transfected with siRNA for 48h before protein isolation, for MTT assays 72h before assessment of cell viability. Cell lines were seeded and, after attachment, transfected with siRNA (GE Healthcare, Freiburg, Germany) to a final concentration of 50 nM ON-TARGETplus Non-targeting Pool, (D-001810-10, referred to as *si* control) or ON-TARGETplus CTNNB1 (L-003482-00, referred to as *si* Δ-catenin), as indicated, using Lipofectamine2000 (Invitrogen Carlsbad, US) according to manufacturer's instructions [49].

### Cell migration and invasion assays

Migration and invasion assays were carried out using the adapted Boyden chamber transwell-system (NeuroProbe, Gaithersburg, USA). 5 × 10^3^ of 34h pre-treated cells were seeded onto 8 μm pore sized membranes into the upper compartment of the chamber without FCS and ICG-001 or vehicle, respectively. For invasion assays membranes were covered with 1:5 diluted matrigel. To attract cells, medium with 10% FCS was loaded into the lower chamber. After incubation for 14h, the upper surface of the membrane was wiped with a scraper, fixed with 1% methanol /1% formaldehyde and stained with 0.5% crystal violet. The relative number of cells that pass the transwell-filter pores were quantified using ImageJ. For each condition 6 wells were loaded and analyzed. All experiments were performed in triplicates. P values < 0.05 were considered as significant and determined by student's t-tests.

### Colony formation assay

For colony formation assay, 5000 cells were plated into 6-well plates in triplicates, treated with different concentrations of ICG-001 (1 μM to 5 μM) or vehicle and incubated for 8 days. Wells were photographed at 2-fold magnification, and the relative colony area was measured using ImageJ as previously described [50]. Three independent experiments were used for calculation. P values < 0.05 were considered as significant and determined by student's t-tests.

### Sphere formation and migration assay

10000 cells were seeded into 0.5% agarose gel-coated 6-wells in DMEM/F-12 supplemented with 20 ng/ml bFGF, 20 ng/ml EGF and 2.5 μM ICG-001 or vehicle. Medium was replaced after 3 days of incubation. After 6 days, tumorspheres were counted in total and according to their size: large (< 0.3 mm), mid-sized (0.1 to 0.3 mm), and small (0.05 - 0.1 mm). For studying tumorsphere migration, 7 - 9 spheres from three independent experiments (d6) were plated in standard culture medium with 10% FCS to allow cells to adhere. Pictures were taken after 2 h, 16 h, and 32 h, as indicated above. The relative distance and the area covered by migrated cells were determined in relation to the respective spheres at time point 2 h. For quantification of the migration distance, three measure points around the sphere were randomly set with an ankle of 120°. Assays were performed with three different cell line passages. P values < 0.05 were considered as significant and determined by student's t-tests.

### Western blotting and used antibodies

For Western Blotting cells of at least three independent biological replicates were treated, as indicated, and lysed with 1xRIPA buffer; Western Blotting and detection of the proteins with specific antibodies was performed as previously described elsewhere and according to standard protocols (REF). Antibodies against Nestin (2393925), CD133 (2438662) and Klf4 (2116008) were purchased from Millipore (Darmstadt, Germany), against alpha-Tubulin (DLN-09992) from Dianova (Hamburg, Germany) and Oct4 (0421) from DCS (Berlin, Germany). Antibodies against cleaved Caspase-3 (ab90437) and GAPDH (ab8245) were purchased from abcam (Cambridge, UK), the anti β-Actin-Peroxidase antibody (AC-15) was purchased from Sigma-Aldrich (Darmstadt, Germany). The self-made antibody detecting Δ-catenin was previously described in [18].

### Quantitative real-time PCR (qPCR)

RNA was isolated using the High Pure RNA Isolation Kit (Roche, Eisenach, Germany) and was reverse transcribed using RevertAid Reverse Transcriptase (Thermo Fisher Scientific, Bremen, Germany) according to manufacturer's instructions. qPCR was carried out according using iQ™ SYBR^®^ Green Supermix (Biorad, München, Germany) and oligonucleotides amplifying *ACTB* (for: 5’-CTGGGAGTGGGTGGAGGC-3’ and rev: 5’-TCAACTGGTCTCAAGTCAGTG-3’), *BMP4* (for: 5’-CTGATGGTCGTTTTATTATGCC-3’ and rev: 5’-CTCAGGTATCAAACTAGCATGG-3’), AXIN2 (for: 5’-TGTGGGCAGTAAGAAACAG-3’ and rev: 5’-CTCGGGAAATGAGGTAGAG-3’), CD44 (for: 5’-ATCCCTCGGGTGTGCTATGGATGG-3’ and rev: 5’-CCTCAGTGGAAAGCAATGCCCAGG-3’), JDP2 (for: 5’-GCTGAAATACGCTGACATC-3’ and rev: 5’-CTCACTTTTCACGGGCTGG-3’) and GADD45A (for: 5’- GAGAGCAGAAGACCGAAAGGA-3’ and rev: 5’- CACAACACCACGTTATCGGG-3’), P values < 0.05 were considered as significant and determined by student's t-tests.

### *Luciferase* (*Luc*) reporter gene assay

Cell lines were seeded and after attachment transfected with 200 ng *Luc*-TOP/FOP reporters and 25 ng CMV-Renilla using Lipofectamine2000 (Invitrogen Carlsbad, US) according to manufacturer's instructions [49]. In addition to ICG-001 and vehicle control treatment, cells were co-incubated with Wnt3a- or control-conditioned media produced in L cells for 48 h (ATCC). After 48 h of incubation, cells were lysed with passive lysis buffer (Qiagen, Hilden, Germany) and Luciferase activity was measured with Berthold Multimode Reader TriStar LB 946 (Berthold, Bad Wildbad, Germany) according to standard protocols and as previously described [18]. P values < 0.05 were considered as significant and determined by student's t-tests.

### FACS analysis

For cell cycle analysis pedHGG cells were incubated with ICG-001 or vehicle for 24 h, trypsinized and fixed in 70% Ethanol for 30 min at 4°C. DNA was stained with propidium iodide (50 μg/ml, Roche, Eisenach, Germany) after RNase treatment (100 μg/ml, AppliChem, Darmstadt, Germany) for 30 min. For the assessment of cell divisions, pedHGG cells were preincubated with CFSE (CFSE Cell Division Tracker Kit, BioLegend, San Diego, US) in addition to ICG-001 or DMSO, respectively, for 24 h according to manufacturer's instructions. After incubation, cells were trypsinized and subjected to FACS analysis. PI- and CFSE-emissions of single cells were measured with the BD LSRII flow cytometer (BD, New Jersey, US). Data were analyzed using FlowJo software (Flowjo, Oregon, US). P values < 0.05 were considered as significant and determined by student's t-tests.

### Chicken chorioallantoic membrane (CAM) assays

CAM assays were performed as described previously [51]. Briefly, 2 × 10^6^ of ICG-001 and vehicle 48h pretreated cells were implanted onto a 10 days old chicken embryo CAM in 40 μl extracellular matrix (ECM) supplemented with ICG-001 or vehicle and incubated for further 7 days. Tumors were analyzed with ImageJ. At least two different cell line passages were used. Overall, at least 10 grown tumors per condition and cell line were analyzed. P values < 0.05 were considered as significant and determined by student's t-tests.

### RNA-sequencing and data analysis

RNA was isolated from two independent biological replicates of KNS42 and UW479 cells, and three independent biological replicates of SF188 cells after treatment with ICG-001 or vehicle for 48h using the High Pure RNA Isolation Kit (Roche, Eisenach, Germany) according to manufacturer's instructions. Library preparation and sequencing analysis were performed as described before [52, 53]. Candidate genes were filtered to a minimum of 2x fold changes and FDR-corrected p-value<0.05. For functional analysis, gene ontology enrichment was tested accounting for gene length via R-package goseq. GO-annotation analyses were performed using DAVID Bioinformatics Resources 6.8 (Frederick, US) [28, 54]. Sequence data has been deposited in NCBI's Gene Expression Omnibus and are accessible through GEO Series accession number GSE83266.

### Patients data and survival analysis

Normalized gene expression data from 78 tumor samples of pedGBM patients (age range 3 to 20 years) and corresponding survival data of 61 patients were kindly provided by Stefan Pfister and Dominik Sturm (DKFZ, Heidelberg, Germany; GEO accession numbers: GSE36245, GSE34824, GSE19578). Subgroups of pedHGG patients of the indicated genes were defined as < median gene expression (=low) and ≥ median gene expression (=high) based on the median gene expression from all available samples. Overall survival (OS) was determined by Kaplan-Meier analysis. Statistical significance was defined by p<0.05 as analyzed by log-rank testing. Statistical analyses were performed using STATA software (StataCorp LP, College Station, USA).

## SUPPLEMENTARY MATERIALS FIGURES AND TABLES




