# Supplementary material for: The β-catenin/CBP-antagonist ICG-001 inhibits pediatric glioma tumorigenicity in a Wnt-independent manner
Source: Oncotarget. 2017 Mar 6;8(16):27300–13. doi: 10.18632/oncotarget.15934 (PMC5432336; doi:10.18632/oncotarget.15934)
Supplement: Supplementary file 1 [file oncotarget-08-27300-s001.pdf]

The β-catenin/CBP-antagonist ICG-001 inhibits pediatric glioma tumorigenicity in a Wnt-independent manner

SUPPLEMENTARY DATA

REFERENCES

1. Lee Y, Lee JK, Ahn SH, Lee J, Nam DH. WNT signaling in glioblastoma and therapeutic opportunities. Laboratory investigation. 2016; 96:137-150.
2. Samuel H. Cheshier LA, Irving L. Weissman , Victor Tse, Stephen Skirboll. Activated Canonical Wnt Signaling in GBM is Associated with Increased Expression of Stem Cell Surface Markers. Cureus. 2011; 3: e25.
3. Kahlert UD, Maciaczyk D, Doostkam S, Orr BA, Simons B, Bogiel T, Reithmeier T, Prinz M, Schubert J, Niedermann G, Brabletz T, Eberhart CG, Nikkhah G, Maciaczyk J. Activation of canonical WNT/beta-catenin signaling enhances in vitro motility of glioblastoma cells by activation of ZEB1 and other activators of epithelial-to-mesenchymal transition. Cancer letters. 2012; 325:42-53.
4. Yue X, Lan F, Yang W, Yang Y, Han L, Zhang A, Liu J, Zeng H, Jiang T, Pu P, Kang C. Interruption of beta-catenin suppresses the EGFR pathway by blocking multiple oncogenic targets in human glioma cells. Brain research. 2010; 1366:27-37.
5. He TC, Sparks AB, Rago C, Hermeking H, Zawel L, da Costa LT, Morin PJ, Vogelstein B, Kinzler KW. Identification of c-MYC as a target of the APC pathway. Science (New York, NY). 1998; 281:1509-1512.
6. Karaulanov E, Knochel W, Niehrs C. Transcriptional regulation of BMP4 synexpression in transgenic Xenopus. EMBO J. 2004; 23:844-856.
7. Arensman MD, Telesca D, Lay AR, Kershaw KM, Wu N, Donahue TR, Dawson DW. The CREB-binding protein inhibitor ICG-001 suppresses pancreatic cancer growth. Molecular cancer therapeutics. 2014; 13:2303-2314.

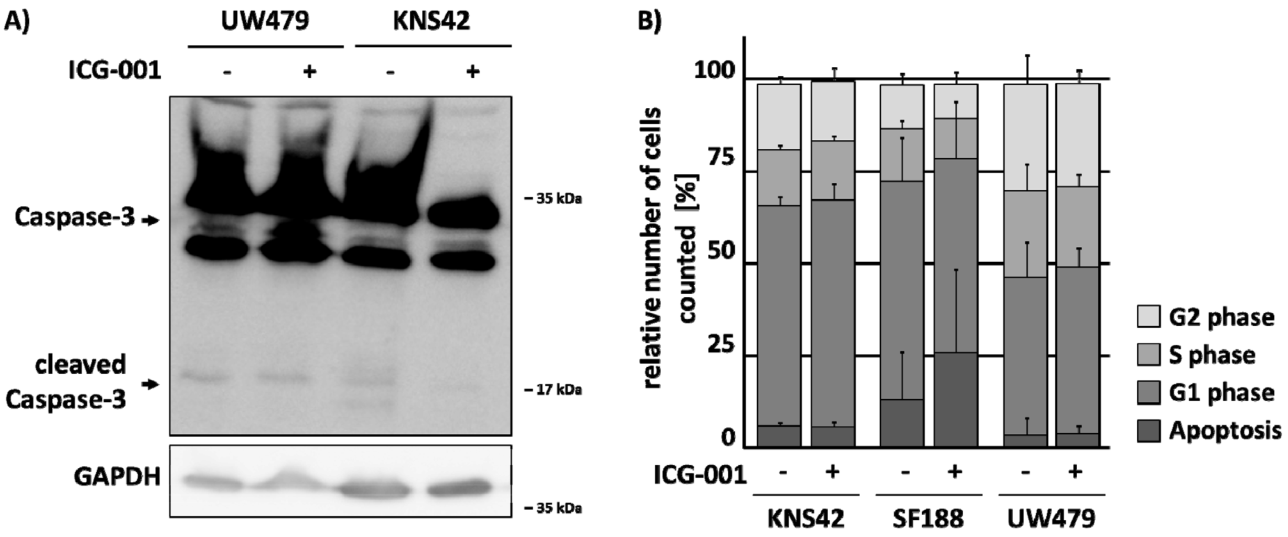

Supplementary Figure 1: ICG-001 treatment does not induce apoptosis and has no impact on cell cycle phase distribution. KNS42, SF188 and UW479 glioma cells treated with ICG-001 or vehicle, respectively, were subjected to **(A)** Western Blot analyses probed with GAPDH and Caspase-3 antibodies detecting full-length and cleaved Caspase-3 to confirm apoptosis and **(B)** propidium iodide-FACS analyses detecting cell cycle phase distribution in comparison to vehicle treated control cells.

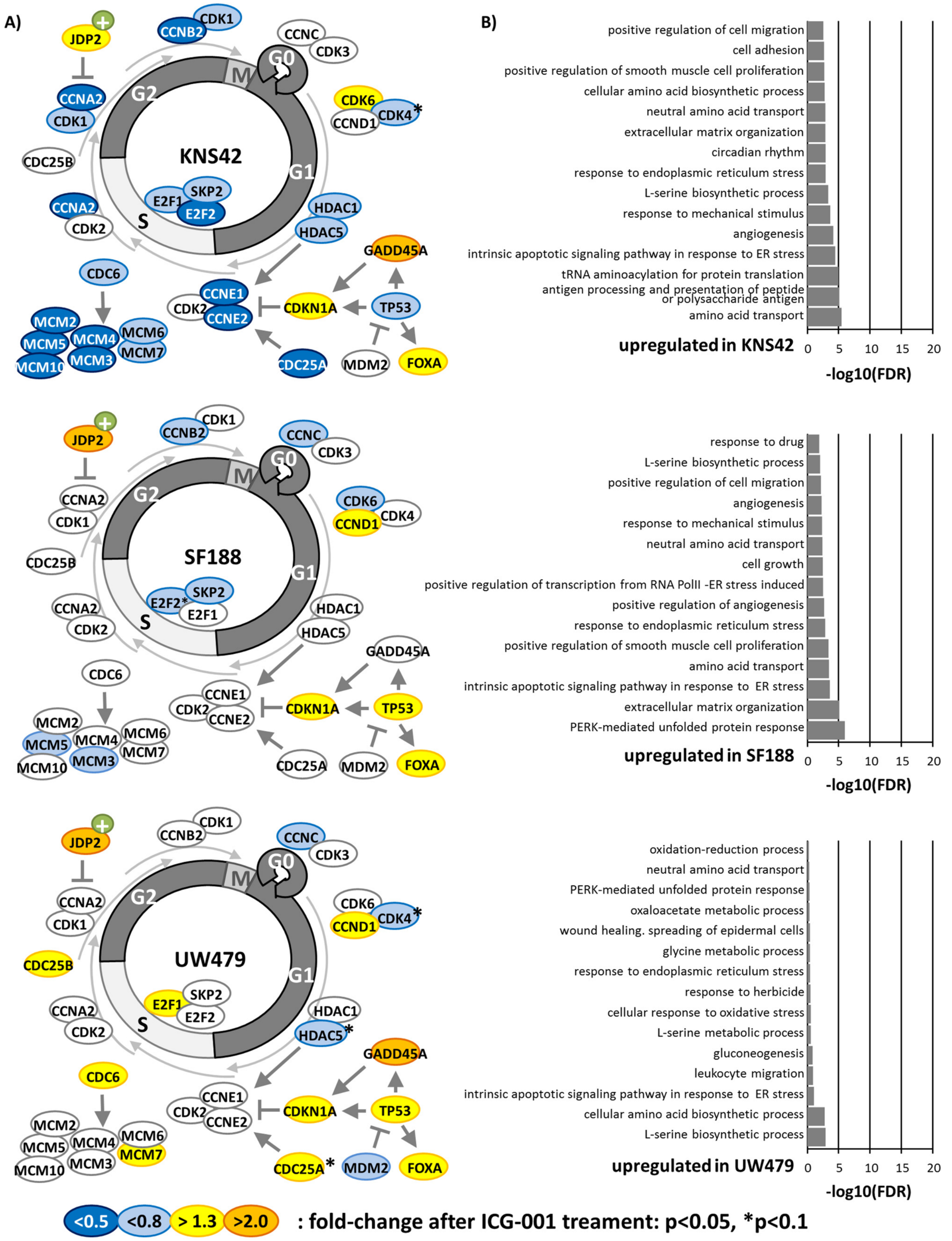

Supplementary Figure 2: Impact of ICG-001 on cell cycle regulating genes and up-regulated GO-annotated pathways. **(A)** Effects of ICG-001 on the expression of cell cycle-regulating key-factors in KNS42, SF188 and UW479 cells, expressional changes are indicated by color codes. **(B)** ICG-001 treatment increases metabolic and biosynthetic processes. GO-enrichment analyses showing the fold discovery rate (FDR) of the top 15 enriched biological processes of all significantly ($p<0.05$), 2-fold upregulated genes after treatment with ICG-001 for 48h in each cell line and commonly emerging GO-annotated processes.

Supplementary Table 1: Base mean and fold changes of canonical β-catenin/Wnt target genes (for details, see references) after ICG-001-treatment in comparison to vehicle-treated KNS42, SF188 and UW479 cells determined by mRNA-sequencing

cell line/ GeneID	KNS42			SF188			UW479			ref.
	base mean	fold change	adjustedp-value	base mean	fold change	adjustedp-value	base mean	fold change	adjustedp-value	
LEF1	626	1.29	0.10	510	1.12	0.45	5	0.99		[1]
TCF7L2	759	1.30	0.17	27	1.62	0.05	721	0.90	0.53	
NOTCH1	3,064	0.58	0.00	1,088	1.08	0.62	393	1.21	0.28	[2]
CD44	59,090	1.78	0.00	17,642	1.42	0.00	5,405	1.04	0.92	
PROM1	74	1.06	0.88	100	1.08	0.83	2	0.97		
EGFR	3,507	2.23	0.00	3,220	1.49	0.00	6,043	1.00	1.00	
FOXM1	5,139	0.70	0.34	3,541	1.09	0.50	7,580	1.69	0.00	[1]
AXIN2	239	1.16	0.54	107	0.71	0.05	0	1.03		
WIF1	-	1.00		0	1.03		-	1.00		
ZEB1	2,253	1.08	0.60	2,792	1.26	0.01	108	0.69	0.13	[3]
SNAI1	38	1.18	0.69	467	0.73	0.01	24	1.34		
CDH2	27,948	1.33	0.00	5,512	1.50	0.00	1	0.97		
CCND1	19	1.15		67,269	1.76	0.00	10,062	1.63	0.00	[4]
AKT1	2,720	1.00	1.00	5,090	0.93	0.46	1,863	1.14	0.40	
JUN	5,665	3.82	0.00	3,596	1.43	0.00	2,001	1.42	0.05	
VEGFA	2,109	11.22	0.00	2,706	3.15	0.00	4,320	1.63	0.00	
MMP9	1	0.94		25	2.93	0.00	49	1.36		
MYC	341	4.14	0.00	15,495	1.17	0.12	2,492	1.33	0.05	[5]
BMP4	142	1.20	0.61	674	0.63	0.00	18	0.93		[6]
EDN1	194	1.96	0.00	33	2.85	0.00	50	0.90		[7]
LGR5	1	1.08		-	1.00		-	1.00		
RNF43	1	1.09		232	0.80	0.14	328	1.18	0.39	
PORCN	789	1.38	0.02	690	1.44	0.00	260	1.38	0.08	

Supplementary Table 2: Base mean and fold changes of cell cycle associated genes after ICG-001 treatment in comparison to vehicle-treated KNS42, SF188 and UW479 cells determined by mRNA-sequencing

See Supplementary File 1
