# Supplementary material for: The β-catenin/CBP-antagonist ICG-001 inhibits pediatric glioma tumorigenicity in a Wnt-independent manner
Source: Oncotarget. 2017 Mar 6;8(16):27300–13. doi: 10.18632/oncotarget.15934 (PMC5432336; doi:10.18632/oncotarget.15934)
Supplement: Supplementary file 2 [file oncotarget-08-27300-s002.doc]

**SUPPLEMENTAL INFORMATION**

**Supplemental Table 2: Base mean and fold changes of cell cycle associated genes after ICG-001 treatment in comparison to vehicle-treated KNS42, SF188 and UW479 cells determined by mRNA-sequencing.**

| **cell line/**  **Gene ID** | **KNS42** | | | **SF188** | | | **UW479** | | |
| --- | --- | --- | --- | --- | --- | --- | --- | --- | --- |
|  | **base mean** | **fold change** | **adjusted p-value** | **base mean** | **fold change** | **adjusted p-value** | **base mean** | **fold change** | **adjusted p-value** |
| *CCNA1* | 77 | 0.71 | 0.28 | 2 | 1.03 |  | - | 1.00 |  |
| *CCNA2* | 1,293 | 0.35 | 0.00 | 2,295 | 0.90 | 0.24 | 3,900 | 1.07 | 0.71 |
| *CCNB1* | 2,434 | 0.56 | 0.09 | 5,374 | 0.77 | 0.00 | 4,352 | 1.20 | 0.21 |
| *CCNB2* | 1,953 | 0.36 | 0.00 | 2,382 | 0.64 | 0.00 | 4,588 | 1.18 | 0.22 |
| *CCNB3* | 437 | 0.62 | 0.17 | 39 | 0.90 | 0.76 | 160 | 0.80 | 0.37 |
| *CCNC* | 2,233 | 0.83 | 0.19 | 904 | 0.79 | 0.02 | 2,563 | 0.71 | 0.01 |
| *CCND1* | 19 | 1.15 | - | 67,269 | 1.76 | 0.00 | 10,062 | 1.63 | 0.00 |
| *CCND2* | 4,017 | 1.10 | 0.73 | 145 | 1.22 | 0.44 | 2 | 1.15 | - |
| *CCND3* | 1,639 | 0.86 | 0.35 | 1,664 | 0.91 | 0.53 | 5,651 | 1.12 | 0.44 |
| *CCNE1* | 179 | 0.45 | 0.00 | 583 | 0.98 | 0.93 | 477 | 1.18 | 0.33 |
| *CCNE2* | 413 | 0.47 | 0.00 | 344 | 1.07 | 0.74 | 286 | 1.02 | 0.95 |
| ***CDKN1A^2^*** | **366** | **1.40** | **0.08** | **642** | **1.44** | **0.00** | **2,636** | **1.45** | **0.01** |
| *CDKN2A* | 1,830 | 1.23 | 0.36 | 7,155 | 1.12 | 0.58 | - | 1.00 | - |
| *CDK1* | 2,180 | 0.51 | 0.00 | 3,728 | 0.91 | 0.38 | 3,978 | 1.27 | 0.12 |
| *CDK2* | 2,901 | 0.92 | 0.61 | 2,658 | 0.91 | 0.38 | 2,896 | 1.16 | 0.26 |
| *CDK4* | 3,288 | 0.65 | 0.00 | 115,006 | 0.99 | 0.95 | 2,856 | 1.31 | 0.10 |
| *CDK6* | 3,274 | 1.39 | 0.01 | 3,451 | 1.39 | 0.01 | 1,430 | 0.82 | 0.17 |
| *CDC6* | 2,215 | 0.52 | 0.00 | 3,182 | 1.12 | 0.42 | 2,015 | 1.39 | 0.01 |
| *CDC7* | 1,257 | 0.72 | 0.01 | 1,195 | 1.00 | 1.00 | 1,056 | 1.12 | 0.48 |
| *MCM2* | 5,117 | 0.44 | 0.00 | 6,414 | 0.85 | 0.15 | 4,002 | 1.09 | 0.53 |
| *MCM3* | 8,052 | 0.38 | 0.00 | 5,192 | 0.78 | 0.03 | 4,830 | 1.02 | 0.89 |
| *MCM4* | 5,752 | 0.49 | 0.00 | 8,370 | 0.91 | 0.50 | 5,353 | 0.86 | 0.32 |
| *MCM5* | 1,782 | 0.33 | 0.00 | 5,336 | 0.78 | 0.02 | 3,577 | 1.02 | 0.92 |
| *MCM6* | 5,295 | 0.55 | 0.00 | 4,391 | 0.79 |  | 3,369 | 0.89 | 0.38 |
| *MCM7* | 11,756 | 0.79 | 0.02 | 15,636 | 1.01 | 0.95 | 4,498 | 1.65 | 0.00 |
| *MCM8* | 2,060 | 0.78 | 0.03 | 3,466 | 1.07 | 0.63 | 1,206 | 1.20 | 0.22 |
| *MCM9* | 764 | 0.96 | 0.86 | 231 | 1.10 | 0.60 | 362 | 0.90 | 0.62 |
| *MCM10* | 849 | 0.45 | 0.00 | 2,651 | 0.95 | 0.77 | 1,061 | 1.24 | 0.15 |
| *GADD45A* | 659 | 2.10 | 0.00 | 988 | 1.17 | 0.19 | 1,089 | 2.15 | 0.00 |
| *TP53* | 1,236 | 0.69 | 0.00 | 5,709 | 1.01 | 0.94 | 3,012 | 1.52 | 0.00 |
| *CDC25A* | 605 | 0.46 | 0.00 | 908 | 0.98 | 0.93 | 820 | 1.31 | 0.10 |
| *CDC25B* | 9,212 | 1.12 | 0.60 | 6,979 | 1.04 | 0.78 | 4,542 | 1.63 | 0.00 |
| *CDC25C* | 144 | 0.61 | 0.13 | 429 | 0.74 | 0.01 | 1,063 | 1.12 | 0.49 |
| *CDC37* | 3,998 | 0.63 | 0.00 | 5,900 | 0.88 | 0.25 | 3,908 | 1.02 | 0.88 |
| *E2F1* | 1,370 | 0.52 | 0.00 | 2,038 | 0.89 | 0.39 | 2,364 | 1.50 | 0.01 |
| *E2F2* | 477 | 0.38 | 0.00 | 471 | 0.77 | 0.09 | 1,116 | 1.21 | 0.18 |
| *HDAC1* | 4,327 | 0.58 | 0.00 | 3,914 | 0.90 | 0.30 | 3,811 | 0.84 | 0.18 |
| *HDAC5* | 2,454 | 0.73 | 0.05 | 2,226 | 0.85 | 0.12 | 1,131 | 0.76 | 0.07 |
| *MDM2* | 2,112 | 0.91 | 0.58 | 1,531 | 1.05 | 0.77 | 2,105 | 0.74 | 0.03 |
| *SKP2* | 2,508 | 0.52 | 0.00 | 1,482 | 0.78 | 0.01 | 1,375 | 0.82 | 0.19 |
| ***JDP2^1/2^*** | **248** | **5.09** | **0.00** | **315** | **10.17** | **0.00** | **1,194** | **1.54** | **0.00** |
| ***PMAIP1^2^*** | 3,654 | 1.77 | 0.00 | 4,304 | 1.66 | 0.00 | 201 | 1.57 | 0.04 |

***^1^*** *correlated with pediatric patients’ survival*

***^2^*** *commonly regulated in KNS42, SF188 and UW479 cells*
